# How the desert ant *Cataglyphis fortis* uses its nest hill for homing

**DOI:** 10.1242/jeb.252456

**Published:** 2026-05-26

**Authors:** Lin Jiang, Markus Knaden

**Affiliations:** Department of Evolutionary Neuroethology, Max Planck Institute for Chemical Ecology, 07745 Jena, Germany

**Keywords:** Visual cues, Landmark navigation, Cue integration, Multimodal navigation

## Abstract

The desert ant *Cataglyphis fortis* relies on path integration and environmental cues for navigation. In featureless environments, nest hills serve as landmarks that enhance homing success. We found that homing ants can discriminate their own nest hills from others based on visual cues. When, as a result of experimental displacement, the path integrator points towards a foreign nest, the ants compare visual cues from the perceived nest hill with their memory and continue approaching only if the hill size does not deviate markedly from the memorised one. Homing ants that mistake a foreign hill for their own climb it, indicating that the hills do not provide nest-specific contact cues. However, near the nest entrance in the centre of the volcano-shaped hills, nest-specific cues circumvent the risk of ants entering the wrong nest, where they would be killed. These findings demonstrate the integration of different modalities that enable ants to optimise their homing.

## Teaser

**Highlighted Article:** Desert ants take their nest hills as visual cues and discriminate them from the hills of foreign nests.

## INTRODUCTION

The desert ant *Cataglyphis fortis* inhabits the hostile, featureless salt pans of Tunisia. Its individual foragers have evolved a highly efficient navigational system that allows them to return safely and rapidly to the nest after foraging over hundreds of metres (Knaden, 2019; Knaden and Graham, 2016). *Cataglyphis* ants do not use pheromone trails; path integration (PI) is the predominant navigational strategy, relying on a sun compass and a step counter to continuously update the ants' position relative to the nest entrance (Wehner, 2008; Wehner and Müller, 2006; Wittlinger et al., 2006). As PI accumulates error with increasing foraging distance (Merkle et al., 2006), ants additionally learn and use geocentric cues, such as visual (Wehner et al., 1996) and olfactory information (Buehlmann et al., 2012, 2015; Wolf and Wehner, 2000), for navigation.

Ants have been shown to actively scan the environment for visual landmarks (Clement et al., 2025) and before the first foraging trips, novices learn nest-centred panoramic views through a series of learning walks around the nest (Fleischmann et al., 2017, 2018; Freas et al., 2019). Panorama learning continues during subsequent foraging, along both outbound and inbound routes. Ants retain these visual cues in long-term memory, and when away from the nest, they compare their current view with stored views, homing in the direction that minimises mismatch (Baddeley et al., 2012; Collett et al., 2001, 2006; Freas et al., 2019; Zeil et al., 2014).

In visually sparse environments, desert ants build nest hills that serve as landmarks to locate their nests more efficiently. Nests on flat salt pans have significantly taller nest hills than those in structured environments (Freire et al., 2023). Here, we asked how the ants use their nest hills for homing. (i) Can ants discriminate their own nest from others based on the nest hill? (ii) Do nest hills provide contact information in addition to visual cues? (iii) Do ants enter the entrances of foreign nests during homing?

## MATERIALS AND METHODS

### Experimental site and ant species

The model organism of this study was the desert ant *Cataglyphis fortis* (Forel 1902). Field experiments were conducted between 20 June and 13 July 2025, from 07:00 h to 14:00 h, in a salt pan near the village of Menzel Chaker in Tunisia (Sebkhet Bou Jemel, coordinates: 34°58′N 10°25′E).

### Procedure

**Fig. 1.**
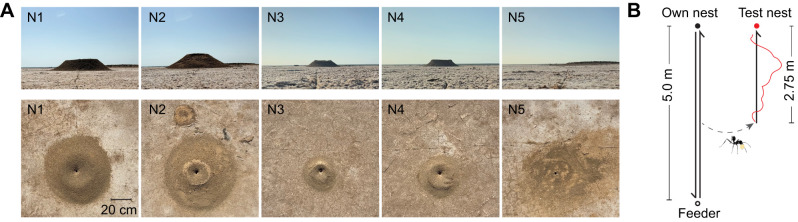
**Experimental design.** (A) Side and top profiles of five nests (N1–N5), classified by the presence and size of their hills: large (N1, N2), small (N3, N4) and absent (N5). Photos of side profiles are taken from the position where the ants were released (2.75 m from the nest). (B) Schematic diagram of the experimental procedure. Ants were trained to a feeder 5.0 m from their nest. Returning foragers were captured 2.75 m from the entrance and released either at the same distance from their own nest or at an equivalent distance and direction from a foreign nest. Their homing paths were tracked following release.

To investigate whether *C. fortis* ants can discriminate their nest from others based on the nest hill, we first selected five nests: two had similar large hills, two had similar small hills, and the hill of the remaining nest was removed 2 weeks before the behavioural experiment started (Fig. 1A). The morphometric parameters of each nest hill were measured using a 30 cm ruler (Table 1). We also obtained the corresponding geographic coordinates using a GPS tracker and Google Maps.

**Table 1.** Morphometric parameters of nest hills

Nest	Hill diameter (cm)		Hill height (cm)	Geographic coordinates
	Base	Top		
N1	65	29	13	34°57'25.6″N, 10°24'34.4″E
N2	70	22	15	34°57'26.0″N, 10°24'32.7″E
N3	35	18	9	34°57'28.4″N, 10°24'35.5″E
N4	38	20	8	34°57'28.4″N, 10°24'35.9″E
N5	—	—	—	34°57'24.9″N, 10°24'33.1″E

Note: the hill of nest 5 was experimentally removed prior to the behavioural test.

**Fig. 2.**
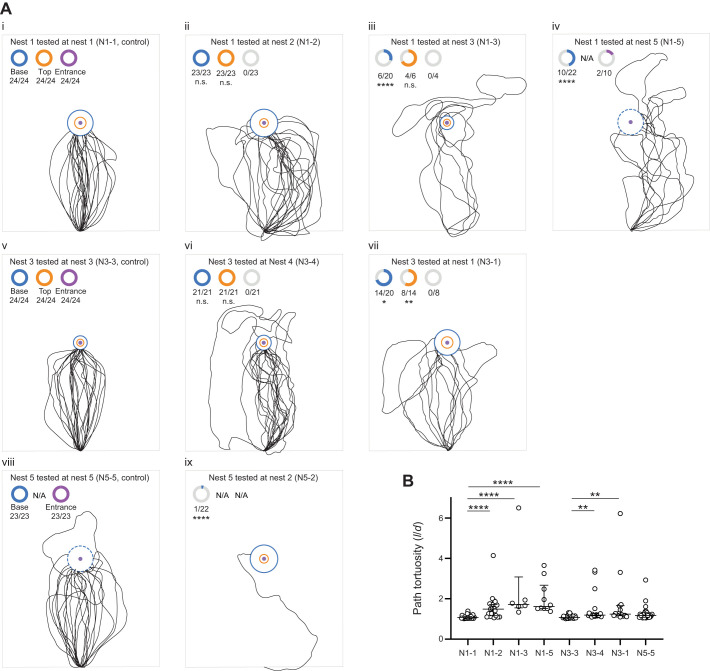
**Ant homing performance under different experimental conditions.** (A) Homing trajectories and success rates to reach the base of the hill (outer blue circle), the top of the hill (inner orange circle) and the nest entrance (central purple dot). The dashed circle around N5 represents an estimated hill base, as the original nest hill had been removed prior to the experiment. (Ai,v,viii) Ants navigating toward their own nest (control). (Aii,vi) Ants navigating toward a foreign nest with a similar hill. (Aiii,vii) Ants navigating toward a foreign nest with a different hill. (Aiv,ix) Ants navigating toward a foreign nest without a hill or with a hill. Only individuals that reached the hill base are displayed (see Fig. S1 for all recorded trajectories). Doughnut charts summarise the success rate of ants reaching each location: the hill base (blue), the hill top (orange) and the nest entrance (purple). For each treatment, the two success rates (at the base and top of the hills) of ants tested at a foreign nest were compared with the corresponding success rates of the control experiment at their own nest (left panels). Fisher's exact tests with Bonferroni correction for multiple comparisons between selected pairs: n.s., *P*>0.05; *P<0.05; **P<0.01; ****P<0.0001. N/A indicates that the metric was not applicable for statistical analysis in the hill-absent group (iv, N1–5 and viii, N5-5) for the top location. N/A is also shown in the group where only one out of 22 tested ants reached the base of the foreign nest and did not proceed to climb, as it was excluded from the analysis (ix, N5-2). (B) Path tortuosity [i.e. run length (*l*) divided by straight-line distance (*d*)] of homing ants reaching the base of different nest hills; bars represent medians±interquartile range. There was no significance difference between the three control groups (N1-1, N3-3, N5-5, returning to their own nests). Kruskal–Wallis test with Dunn's multiple comparisons test for selected pairs: n.s., *P*>0.05; **P<0.01; ****P<0.0001. Because group N5-2 contained only data from a single ant, it was excluded from the analysis. For corresponding raw data, see Dataset 1.

Around each nest, we drew a 4×5 m grid (1 m×1 m cells) on the ground for reference. For each nest, a feeder pit was dug 5 m from the nest entrance always in the same direction and filled with cookie crumbs, such that the paths from the feeder pits to their corresponding nest entrances were parallel. Before the experiment, ants were allowed to travel back and forth between the nest and the feeder for at least 1 h to learn the foraging route and to ensure a sufficient number of active foragers. Returning foragers were then captured at a distance of 2.75 m from the nest entrance. They were released either at the same distance from their nest (see Movie 1) or from a foreign nest at an equivalent distance and direction (Fig. 1B). With reference to Collett et al. (1992), we followed these homing ants several metres behind and manually recorded their paths by drawing them on squared paper, where 2 cm squares on the paper represent 1 m squares on the ground. Trajectories were recorded from the release site until any of the following occurred: the ant entered the nest entrance, contacted resident ants or reached the boundary of the ground grid. Each ant was tested individually and only once. As the ants were not marked prior to the experiments, we cannot completely exclude the possibility that some individuals tested at their own nest were subsequently tested again, as they disappeared directly into their nest entrance after testing. However, the large foraging population at each tested nest (>100 active foragers) makes repeated testing of the same individuals unlikely. At the same time, ants tested at a foreign nest either entered the nest (and were probably killed inside; see Buehlmann et al., 2012) or stayed outside and were excluded from further experiments). Experimental groups were labelled as e.g. N1-1, N1-2, etc., where the first number refers to the nest the tested ant originates from and the second number denotes the nest at which the ant is then tested. Because of time restrictions, we were not able to run experiments in all possible combinations, and therefore had to focus on those comparisons shown in Fig. 2A.

### Quantification and statistical analysis

Ant trajectories were manually digitised in Adobe Illustrator (version 29.8.1, Adobe Inc., San Jose, CA, USA) using the pen tool. For each trajectory, the total path length was directly obtained from the software after selecting the digitised path. Based on the measured nest hill parameters, the basal and upper boundaries of each hill were plotted to scale on the digitised map (shown as concentric circles in Fig. 2A).

Path tortuosity (*l*/*d*) was calculated as the ratio of the path length (*l*) travelled by a homing ant from the release point to the base of the hill to the straight-line distance (*d*) between these two points, thus reflecting path straightness rather than local path meander. Because both *l* and *d* were extracted from the same digitised map, their ratio was independent of the measurement unit. As the original large nest hill of nest 5 was removed prior to the experiment without measurement, we used an estimated base diameter, intermediate between those of N1 and N2, for the analysis in N5-5. For N1-5, we used the nest hill diameter of N1, as ants from N1 were expected to navigate based on the memorised size of their own nest hill.

The success rates for reaching either the base or the top of the nest hill and for entering the nest were calculated as percentages. For nest entry, only ants that actively entered the nest entrance without any contact with resident ants were considered.

For each treatment, the two success rates (at the base and top of the hills) of ants tested at a foreign nest were compared with the corresponding success rates of the control experiment at their own nest (left panels in Fig. 2A) using Fisher's exact tests with Bonferroni correction for multiple comparisons between selected pairs. Path tortuosity of an ant approaching the nest hill reflects the use of visual cues. More direct paths (lower *l*/*d* ratios) indicate confident orientation, whereas increased tortuosity suggests uncertainty, which may arise when ants encounter unfamiliar or mismatched nest hill cues. Accordingly, differences in path tortuosity were assessed using the Kruskal–Wallis test across all treatment groups excluding N5-2 (because of restricted sample size), followed by Dunn's test for multiple-comparisons for selected pairwise comparisons.

## RESULTS AND DISCUSSION

### Ants can discriminate between their own nest and foreign nests over distance

We first compared the performance of ants returning to their own nests (Fig. 2Ai,v,viii). As expected, all ants returned to their nest from the release point to the nest entrance without any pauses along the way. To further quantify differences in their homing paths, we analysed path tortuosity using the Kruskal–Wallis test, followed by Dunn's *post hoc* comparisons (see Table S1). Overall differences among groups were significant (*H*=68.49, d.f.=7, *P*<0.0001). However, no significant differences were found among the control groups (Fig. 2B, N1-1, N3-3 and N5-5, *P*>0.05), indicating that the presence and size of the hill did not affect the ants' performance when returning to their own nest from a short distance.

We subsequently examined hill base success rates of ants tested at a foreign nest and compared them with those in the control experiment at their own nest (Table S2). For ants originating from nests with a hill and tested at a foreign nest with a similarly sized hill, all individuals arrived at the hill base (Fig. 2Aii,vi). However, when the hill of the foreign nest was smaller or larger than their own, the success rate (Fig. 2Aiii,vii) decreased significantly as compared with the control experiment (Fig. 2Ai,v). When ants originating from a nest with a hill were tested at a nest without a hill, 45% of the individuals reached the expected location of the nest hill base (Fig. 2Aiv). At the same time, of those ants originating from a nest without a hill that were displaced close to a nest with hill, only one individual of 22 tested ants arrived at the base of the foreign nest, whereas the others avoided the area (Fig. 2Aix).

In conclusion, ants approached foreign hills whenever they had a similar size to their own hill, even if they slightly differed in their shape (compare hill shapes of N1 versus N2 and N3 versus N4 in Fig. 1A).

Did the ants ignore the differences in shape entirely (for shape differences compare, for example, the similar sized N1 and N2 in Fig. 1A)? To address this question, we compared the tortuosity of their homing paths. The tortuosity of the path toward a foreign nest was significantly higher than that of ants returning to their own nest, even when the size of the foreign nest's hill was similar (Fig. 2B). It seems that ants can identify their own nests from foreign nests at a distance based on the visual characteristics of the nest hill. However, despite being aware of the shape differences, the ants continued to approach the hill of a foreign nest as long as its size matched the remembered size.

Navigation is often achieved through the integration of cues from multiple sensory modalities. Not only are ants known to employ, for example, PI and visual cues simultaneously, but also they are capable of weighting these cues optimally based on their reliability (Buehlmann et al., 2017; Knaden and Graham, 2016; Legge et al., 2014; Wystrach et al., 2015). Our experiments focus on how confident ants are in their nest hill when its visual features are altered. The increased path tortuosity of ants when tested at a foreign hill indicates the ants' internal recognition of a mismatch between current visual cues and their memory. When the mismatch between the ant's perception of a hill and its memory of the hill increases (e.g. when the hill is too small or too big), the ant will reject the hill, even if its PI points towards it. When the mismatch is less significant yet still detectable (e.g. the hill is the right size but slightly different in shape), the PI, despite being affected (see increased tortuosity in Fig. 2B) finally overrides the ant's hesitation caused by the mismatch, prompting it to approach the hill.

### Nest hills do not contain any nest-specific contact cues

Next, we asked whether hills, beyond the visual cues, might also provide colony-specific contact information. Upon reaching their nest hill, ants must climb to the top to reach the entrance to their nest, which is located in the centre of the volcano-shaped hill. We analysed hill top success rates of ants tested at a foreign nest and compared them with those in the control experiment at their own nest (Table S3). When tested on a foreign hill of a similar size to their own, all of the ants climbed to the top (see Fig. 2Aii,vi). Interestingly, when the size of the foreign hill did not match that of the ants' own hill, not all of the ants climbed to the top (Fig. 2Aiii,vii). In particular, ants encountering a larger-than-expected hill (Fig. 2Avii) were significantly more likely to stop climbing as compared with the control (Fig. 2Av).

Ants are known to use the tactile cues, i.e. the structure of the ground, as landmarks (Seidl and Wehner, 2006). At the same time, other ant species have been shown to mark the close vicinity of their nest entrance with gustatory cues that in some cases can be colony specific (Cammaerts and Cammaerts, 1998, 1999; Mayade et al., 1993). We hypothesised that the ants in our experiments would differentiate a foreign hill from their own based on tactile or gustatory cues and refuse to climb it. However, ants continued to climb foreign nest hills, indicating that the hills do not convey nest-specific contact cues. Ants are known to perform PI in three dimensions (Wohlgemuth et al., 2001) but to store this information in only a two-dimensional home vector (Grah et al., 2007), i.e. although the ants take uphill and downhill movements into account when calculating their home vector, they do not seem to store the information on how much they have moved uphill or downhill. Our finding that ants that normally only have to climb a small hill to get home, give up when faced with a larger hill (Fig. 2Avii) suggests that ants do have some idea of how high they normally have to climb. However, some of the ants tested on a smaller hill did not reach the top either (Fig. 2Aiii). In other words, they gave up before reaching their usual height. The weaker performance when climbing a hill that is the wrong size might, therefore, be due to the ants' perception of visual input while climbing.

### Nest hill context influences ant behaviour at foreign nest entrances

We finally examined whether ants entered the entrances of foreign nests. After reaching the top of a foreign hill, ants did not actively enter the entrance, regardless of the morphology of their original nest hill (Fig. 2Aii,iii,vi,vii). Because of their close proximity to the entrance, some individuals inevitably encountered resident ants, resulting in aggressive fights. Notably, individuals that did not contact resident ants always paused at the entrance edge, exhibited hesitation and then retreated. In several cases, ants made repeated attempts to enter the entrance; however, each attempt ended at the entrance edge until they were attacked by resident ants and pulled into the nest. By contrast, when ants from nests with a hill were tested in a foreign nest without a hill (Fig. 2Aiv), 2 of 22 individuals actively entered the nest entrance.

The risk of aggression increases as ants approach the entrance of a foreign nest (Knaden and Wehner, 2003), and attacks can result in severe or even fatal consequences (Buehlmann et al., 2012). Therefore, discrimination of their own nest from foreign nests is essential. In the study by Buehlmann et al. (2012), when nest-specific visual cues were excluded (i.e. neither the original nest nor the test nest had a hill), ants tended to approach the nest from the downwind direction to acquire olfactory information. Similar homing trajectories were observed in our experiments when ants originating from nests with hills were tested at foreign nests without hills (Fig. 2Aiv). Buehlmann et al. (2012) further reported that all ants tested at a foreign nest entered the nest entrance, and subsequently a large proportion were killed. In contrast, in our experiments, although many ants reached the foreign nest entrance, the proportion that ultimately entered it was very low. This discrepancy may be explained by differences in the visual experience of the ants: while in the other study, the ants were tested on artificial arenas, where visual and tactile cues at different nests perfectly matched (Buehlmann et al., 2012), individuals in our study may have detected a mismatch between the memorised hill of their own nest and the scene encountered at the foreign nest. Such a mismatch could prevent the ants from approaching. However, whether this hesitation was driven solely by a visual mismatch or also by nest-specific olfactory cues emanating from the entrance requires further investigation.

Examples of self-built landmarks for navigation are also found in other animals. Leafcutter ants (*Acromyrmex*) construct cylindrical or spherical thatched structures at their nest entrances as homing cues (Moreira et al., 2019). Male fiddler crabs build sand hoods at their burrow entrances to attract females for mating and also use these structures to locate their own burrows (Kim and Christy, 2015). Our data suggest that desert ants *C. fortis* use their self-built nest hills as landmarks and discriminate them from foreign nest hills mainly by visual cues. These findings further demonstrate how self-generated landmarks (Freire et al., 2023) can enhance navigational robustness in environments devoid of natural cues.

## Supplementary Material

10.1242/jexbio.252456_sup1 Supplementary information

Dataset 1. Raw data
