# Supplementary material for: How the desert ant Cataglyphis fortis uses its nest hill for homing
Source: J Exp Biol. 2026 May 26;229(10):jeb252456. doi: 10.1242/jeb.252456 (PMC13286360; doi:10.1242/jeb.252456)
Supplement: Supplementary information [file jexbio-229-252456-s1.pdf]

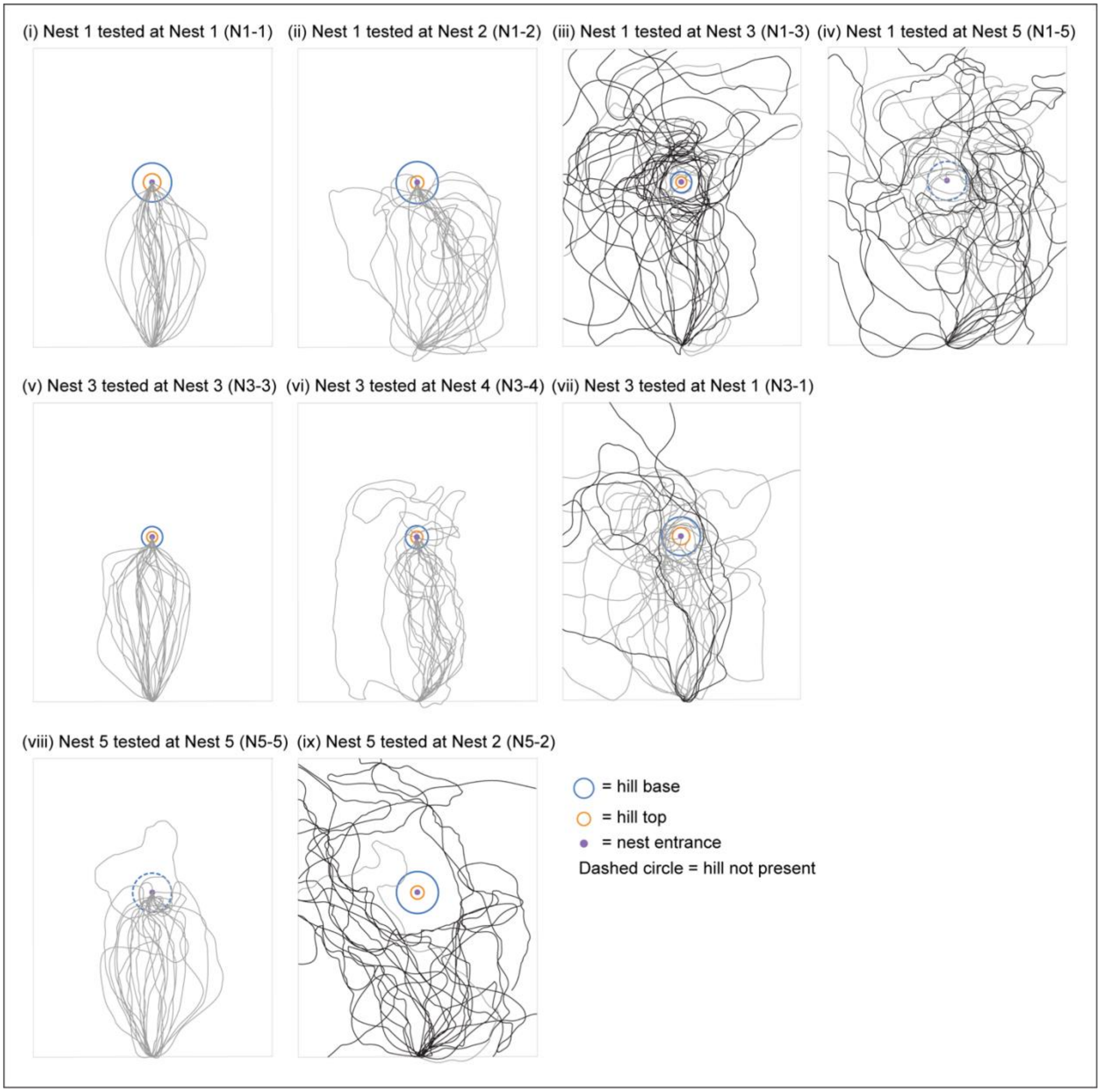

Fig. S1. Complete ant homing trajectories under different experimental conditions.
(i, v, viii) Ants navigating toward their own nest (control). (ii & vi) Ants navigating toward a foreign nest with a similar hill. (iii & vii) Ants navigating toward a foreign nest with a differentially sized hill. (iv & ix) Ants navigating toward a foreign nest without a hill or with a hill.
Trajectories previously illustrated in Figure 2 are shown in grey, whereas additional trajectories in this supplementary figure are shown in black. Trajectories that intersect the purple dot indicate successful nest entry, whereas those that terminate at the entrance boundary indicate that the ants approached but did not enter the nest.

Journal of Experimental Biology • Supplementary information

Table S1. Pairwise comparisons of path tortuosity.

Comparison	Z	Adjusted p-value
N1-1 vs N3-3	0.36	>0.9999
N1-1 vs N5-5	2.77	0.0791
N3-3 vs N5-5	2.41	0.2220
N1-1 vs N1-2	5.00	<0.0001
N1-1 vs N1-3	4.63	<0.0001
N1-1 vs N1-5	5.55	<0.0001
N1-2 vs N1-3	1.42	>0.9999
N1-2 vs N1-5	1.66	>0.9999
N1-3 vs N1-5	0.04	>0.9999
N1-2 vs N3-4	1.13	>0.9999
N1-3 vs N3-1	1.63	>0.9999
N3-3 vs N3-4	3.40	0.0095
N3-3 vs N3-1	3.61	0.0043
N3-4 vs N3-1	0.57	>0.9999

Notes: Overall differences among groups were assessed using the Kruskal–Wallis test (H = 68.49, df = 7, p < 0.0001). Pairwise comparisons were performed using Dunn's test with Bonferroni–Holm correction. Z = test statistic; adjusted p-values are reported.

Table S2. Pairwise comparisons of success rates at the hill base.

Nest	Comparison	Group 1 (Yes/Total)	Group 1 (Yes/Total)	p (Fisher)	p (Bonferroni)
**N1**	**Overall Fisher**	–	–	**5.67×10^{-11}**	–
	N1-1 vs N1-2	24/24	23/23	1.000	1.000
	N1-1 vs N1-3	24/24	6/20	3.37×10^{-7}	1.01×10^{-6}****
	N1-1 vs N1-5	24/24	10/22	1.66×10^{-5}	4.99×10^{-5}****
**N3**	**Overall Fisher**	–	–	**4.69×10^{-4}**	–
	N3-3 vs N3-4	24/24	21/21	1.000	1.000
	N3-3 vs N3-1	24/24	14/20	0.005	0.011*
**N5**	**Overall Fisher (N/A)**	–	–	–	–
	N5-5 vs N5-2	23/23	1/22	5.83×10^{-12}****	N/A

Notes: Success rates of ants tested at a foreign nest were compared with those in the control experiment at their own nest. P-values were obtained using Fisher's exact tests and adjusted for multiple comparisons within each nest using the Bonferroni method. N/A: not applicable (only one comparison in this group). –: not available. * p < 0.05; **** p < 0.0001.

Journal of Experimental Biology • Supplementary information

Table S3. Pairwise comparisons of success rates at the hill top.

Nest	Comparison	Group 1 (Yes/Total)	Group 1 (Yes/Total)	p (Fisher)	p (Bonferroni)
**N1**	**Overall Fisher**	–	–	**0.011**	–
	N1-1 vs N1-2	24/24	23/23	1.000	1.000
	N1-1 vs N1-3	24/24	4/6	0.034	0.069
**N3**	**Overall Fisher**	–	–	**6.67×10^{-5}**	–
	N3-3 vs N3-4	24/24	21/21	1.000	1.000
	N3-3 vs N3-1	24/24	8/14	0.001	0.002**

Notes: Success rates of ants tested at a foreign nest were compared with those in the control experiment at their own nest. P-values were obtained using Fisher's exact tests and adjusted for multiple comparisons within each nest using the Bonferroni method. ** $p < 0.01$. Nest 5 was excluded from the analysis because no hill was present.

Dataset 1. Raw data

Available for download at

https://journals.biologists.com/jeb/article-lookup/doi/10.1242/jeb.252456#supplementary-data

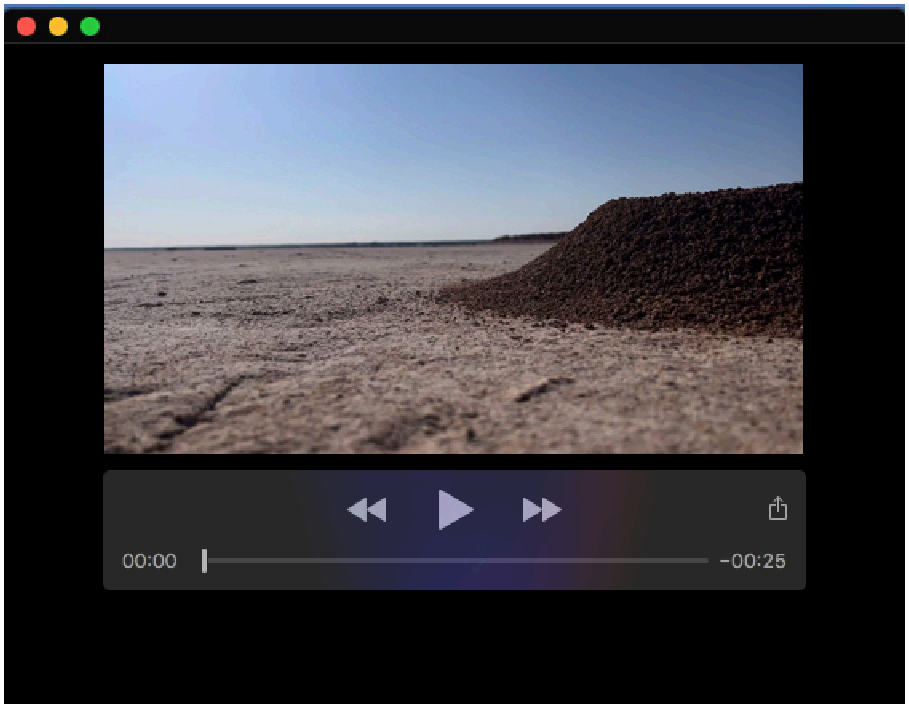

Movie 1. Ant from Nest1 returning to its own nest.
